# Measurement of the Ecological Integrity of Cerrado Streams Using Biological Metrics and the Index of Habitat Integrity

**DOI:** 10.3390/insects8010010

**Published:** 2017-01-12

**Authors:** Deusiano Florêncio dos Reis, Ayala Eduardo Salazar, Mayana Mendes Dias Machado, Sheyla Regina Marques Couceiro, Paula Benevides de Morais

**Affiliations:** 1 Doctorate Program for Biodiversity and Biotechnology of Legal Amazon (ReserachNet BIONORTE), Federal University of Tocantins, Palmas-TO 77001-923, Brazil; deusiano@uft.edu.br; 2 Laboratory for Environmental Microbiology and Biotechnology, Federal University of Tocantins, Palmas-TO 77001-923, Brazil; ayalasalazar@hotmail.com (A.E.S.); mayanamdm@hotmail.com (M.M.D.M.); 3 Laboratory for Ecology and Aquatic Insect Biology, Institute of Water Sciences and Technology, Federal University of West Para, Santarém-PA 68040-050, Brazil; sheylacouceiro@yahoo.com.br

**Keywords:** macroinvertebrates, BMWP, HII, EPT, functional groups

## Abstract

Generally, aquatic communities reflect the effects of anthropogenic changes such as deforestation or organic pollution. The Cerrado stands among the most threatened ecosystems by human activities in Brazil. In order to evaluate the ecological integrity of the streams in a preserved watershed in the Northern Cerrado biome corresponding to a mosaic of ecosystems in transition to the Amazonia biome in Brazil, biological metrics related to diversity, structure, and sensitivity of aquatic macroinvertebrates were calculated. Sampling included collections along stretches of 200 m of nine streams and measurements of abiotic variables (temperature, electrical conductivity, pH, total dissolved solids, dissolved oxygen, and discharge) and the Index of Habitat Integrity (HII). The values of the abiotic variables and the HII indicated that most of the streams have good ecological integrity, due to high oxygen levels and low concentrations of dissolved solids and electric conductivity. Two streams showed altered HII scores mainly related to small dams for recreational and domestic use, use of Cerrado natural pasture for cattle raising, and spot deforestation in bathing areas. However, this finding is not reflected in the biological metrics that were used. Considering all nine streams, only two showed satisfactory ecological quality (measured by Biological Monitoring Working Party (BMWP), total richness, and EPT (Ephemeroptera, Plecoptera, and Trichoptera) richness), only one of which had a low HII score. These results indicate that punctual measures of abiotic parameters do not reveal the long-term impacts of anthropic activities in these streams, including related fire management of pasture that annually alters the vegetation matrix and may act as a disturbance for the macroinvertebrate communities. Due to this, biomonitoring of low order streams in Cerrado ecosystems of the Northern Central Brazil by different biotic metrics and also physical attributes of the riparian zone such as HII is recommended for the monitoring and control of anthropic impacts on aquatic communities.

## 1. Introduction

Aquatic systems worldwide are being threatened by the expansion of human activities [1], mainly by agriculture, cattle ranching, and urbanization [2]. These activities affect not only the physical environment but also the diversity, structure, and function of aquatic communities that thrive in these environments. Tropical freshwater ecosystems are especially at risk of becoming increasingly imperiled by escalating anthropogenic impacts. Nonetheless, they are understudied and not well understood relative to temperate systems [3].

With the exception of some remote Amazonian rivers, most aquatic environments have been altered by human activities in Brazil [4], including wetland drainage, dams, road building, and deforestation for human settlements and intensive agriculture. All of these modifications have a negative impact on the hydrology, vegetation cover, and terrestrial-aquatic linkages of the affected systems [5], but there is a shortage of studies focusing on the consequences of these impacts to ecosystem integrity in Cerrado and Amazonia watersheds.

The Brazilian Savannah (Cerrado) is considered a biodiversity hotspot for conservation priorities [6]. The Cerrado biome is one of the most threatened ecosystems in Brazil. Especially in the Northern part, covered by the Tocantins and Araguaia hydrological region that supports the hydrological forces that link Amazonia production of waters and the Southeast Brazil [7]. The Northern part of the Cerrado biome corresponds in great part to the State of Tocantins whose mosaic of ecosystems characterize the transition from Amazonia to Cerrado. Its functioning and diversity is almost unknown, although it faces the threat of fast occupation due to the creation of the state in the 1990s and the mechanized agriculture and huge damming projects in the Tocantins river mainly, but also in other tributaries.

Vast ecosystems of the Cerrado occupied by the newest Brazilian state have been suffering intense changes in land use [8], mainly due to large-scale soybean agriculture and pasture establishment [9]. In this newly occupied territory the degradation of the riparian stream zone, as well as loss of connectivity to downstream ecosystems, derives mainly from the damming of streams and rivers, often with the purpose of storing water for cattle, together with fast deforestation that threatens the biological integrity of river networks [10]. However, the consequences on the structure of stream ecosystems have not been investigated in these fragile transition ecosystems between the Cerrado and Amazonia biomes. In the Tocantins and Araguaia river basins, large-scale deforestation has contributed to a 25% increase in river flow [11]. In upper Xingu watersheds, covered by plantations in Brazilian Mato Grosso state, Hayhoe et al. [9] reported a reduction in evapotranspiration as well as an increase in flow and seasonal variability compared to forested watersheds; this pattern could be mirrored in the agriculture-dominated landscapes of the Northern Brazilian Cerrado, causing important alterations in regional hydrology. In the neighboring biome, the Brazilian Amazonia, studies conducted in the Madeira River, Ji-Paraná, and upper Jamari basins showed that replacing riparian forest with pastures for grazing affects the hydrology, nutrient concentrations, and benthic habitats of streams. Pasture presence is a major factor affecting the chemical composition of waters [12,13], creating an increase in runoff and lowering habitat complexity from a channel composed of runs and pools and forest leaf detritus (50% cover) to a channel covered with grass (63%), mainly with low-moving waters [14]. Given the steady increase in deforestation in the different ecosystems of the Cerrado in the Tocantins and Araguaia river basins and the fundamental role of its network of rivers in linking the Amazonia to the Southern Cerrado and Atlantic Rain Forest, there is an urgent need for monitoring indicators for the management of these ecosystems.

Among aquatic communities, macroinvertebrates stand out as biological indicators of freshwaters [15,16] since they respond to longer temporal and spatial impacts than the instant measures of physical and chemical variables due to their long-lasting life cycle in waters [17,18]. Also, they show less mobility than larger vertebrates such as fish, as they depend on drifting to move from the habitat if conditions are not suitable.

The use of aquatic macroinvertebrates to assess water quality includes a variety of biotic indexes [19]. The main biotic indices include the presence of sensitive species such as the EPT index (Ephemeroptera, Plecoptera, and Trichoptera) [20] or the adoption of different values of tolerance to organic pollution for each family [21]. Generally, these indices include biological responses in a numeric expression that can be easily understood [22]. They also combine low cost and effort with high efficiency and a fast identification of organisms, even when considering the huge gap of knowledge about biodiversity distribution patterns in tropical areas.

Therefore, this study aimed to evaluate the environmental quality of streams in a partially preserved watershed, the Taquaruçu Grande river basin, using biological metrics based on the macroinvertebrate fauna and abiotic variables such as Habitat Integrity Index. The Taquaruçu Grande river basin can be exemplary of the ecosystems of the mosaic Northern Cerrado territory. It is also facing threats of anthropization, but pristine aquatic environments are still found. The greater threats to this watershed are fast anthropic occupation and touristic activities in rural landscapes, but not organic or chemical pollution derived from urban settlements and agriculture. The effects of these impacts on macroinvertebrate communities and ecosystem health are practically unknown for this territory.

## 2. Materials and Methods

### 2.1. Collection Area

**Figure 1:**
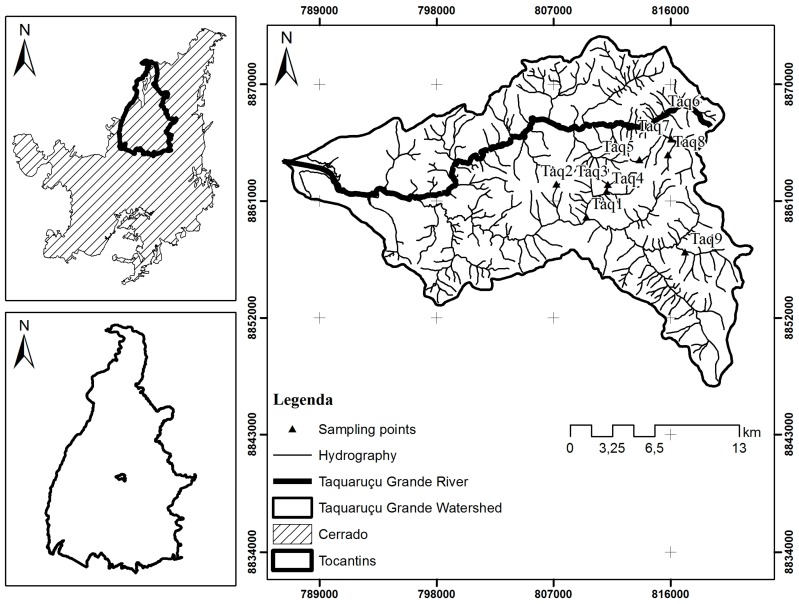
Map of the Taquaruçu Grande river basin showing the sampling points. Source: Bonatto, 2013.

Samples were collected in nine streams located in Taquaruçu Grande river basin (Figure 1). Physical descriptions of the nine streams are presented in Table 1. Taquaruçu Grande is located in the central portion of the state of Tocantins, with 74% of its area of 46,307.31 ha in the Environmental Protection Area of Lajeado (APA Lajeado). Main anthropic pressures are occupation of rural areas by small properties exploring small-scale agriculture, cattle raising, and day-in tourism (activities in waterfalls and bathing areas for 4 to 6 h by neighboring populations), annual fire management of natural pasturelands, and road construction.

Soils are composed of red-yellow latosols (52.93%); red-yellow latosols in association with plinthosol petroferric soil (20.67%); haplic cambisols in association with Lithic neosol (20.34%); red latosols (3.13%); fluvic neosol in association with haplic gleysols (1.23%); plinthosol petroferric soil (1.14%); and lithic neosol (0.56%) [23,24]. Geology of the area consists of lithologies belonging to the domains of the Sedimentary basin of Parnaíba represented by the Pimenteiras and Serra Grande formations from Paleozoic Era and the Lajeado suite from the Neoproterozoic [25]. The vegetation is characteristic of the Cerrado biome and it is represented by gallery forests, seasonal deciduous forests, forested savannas (cerradão), savanna fields (cerrado), and floodplains [26]. Regional climate is tropical savanna rainy climate (Köppen Aw), with two well defined seasons and maximum precipitation in summer (from October to March—1500 mm to 2000 mm), and the dry season in winter (from April to September—less than 60 mm). The average annual temperature is 28 °C, the absolute maximum exceeds 41 °C, and the minimum is higher than 18 °C [25]. The land cover and use of Taquaruçu Grande river basin is comprised of 10% urban area, 23% riparian forest cover, and 55.7% of Cerrado vegetation, with 10% occupied by semi-intensive agricultural and cattle farming production [26]. The upper Taquaruçu Grande basin is characterized by streams with the predominance of runs and rapids, with a wide range of substrate types like gravel, little rocks, and rocks, in a matrix of dense Cerrado vegetation and scattered human occupation.

### 2.2. Macroinvertebrates

Macroinvertebrates were collected in stretches of 200 m in each stream and samplings at ten points 20 m afar, resulting in ten samples per stream during the transition period between dry and rainy season (September–October). All substrates present at the sampling point (leaves, stems, roots, and sediment in both riffles and pools) were collected with a type “D” mesh (0.500 mm mesh size and area of 0.155 m^2^) device. Samples were mixed to form a composite sample per stream.

At the laboratory, the samples were washed with tap water on a 0.25 mm mesh sieve. The macroinvertebrates retained in the sieves were fixed and preserved in ethanol 90%. Identification to family level and counting were carried out using a stereomicroscope (Leica model L2, Leica Microsystems, Mannheim, Germany) and specialized literature) [27,28].

### 2.3. Abiotic Data

The abiotic variables, including temperature, conductivity, pH, total of dissolved solids, and dissolved oxygen, were measured at every sampling point in each stream using a multiparameter probe (Horiba U22-XD model, Horiba Instruments Brasil, Jundiaí-SP, Brazil) resulting in ten measures per sampling effort in each stream. Discharge measurement was taken using a micro-reel hydrometric device (Global Water FT model 111, Global Water, Sacramento, CA, USA) and expressed as the average value of ten measures in each stream.

The physical integrity of the vegetation on the streams margins and its effect on the structure of streams (e.g., sedimentation) was analyzed by Habitat Integrity Index (HII) [29]. This index consists of 12 multiple questions that describe the conditions of the studied area based on visual assessment of land use, the conservation status of the riparian zone, riverbed characteristics, and stream morphology. Each variable has four to six ordered levels according to integrity. The final index varies from zero (more impacted locals) to one (more conserved ones) [29]. We used the quantitative HII to classify the streams in three conservation categories: preserved, altered, and impacted environments, according to field observations. Impacted environments are sites with HII between 0.51 and 0.66. Altered environments have HII between 0.69 and 0.78, and preserved environments have HII varying from 0.82 to 0.96 [30].

### 2.4. Metrics Based on Macroinvertebrates Fauna

In order to represent the ecological quality of streams, nine metrics related to diversity, tolerance/sensitivity, and ecological function of aquatic macroinvertebrates were used. These were: Shannon-Weiner diversity index, Pielou index, richness of macroinvertebrates (number of families), EPT richness (number of families of Ephemeroptera + Plecoptera + Trichoptera), abundance of macroinvertebrates, relative abundance of EPT, *Biological Monitoring Working Party* (BMWP) modified by Monteiro et al. [21], relative abundance of shredders, and relative abundance of detritivores.

As proposed by Monteiro et al. [21], BMWP was used because it presented a better performance for Cerrado aquatic ecosystems among various indexes tested. The classification of macroinvertebrates in shredders and detritivores trophic groups was based on Cummins et al. [31].

### 2.5. Data Analysis

The relation between the abiotic variables, including the HII and metrics based on the macroinvertebrates fauna, were analyzed by Pearson correlations.

## 3. Results

The values of the abiotic variables and of the Habitat Integrity Index are shown in Table 2. Site Taq9 was classified as an impacted environment with a HII of 0.56, due to a surrounding matrix of open pasture and narrow riparian cover together with proximity to a road and suburban area. Site Taq8 was classified as an altered environment with a HII of 0.77, and environmental changes measured in this site refer mainly to changes due to partial removal of marginal vegetation, resulting in a pioneer herbaceous and shrub cover with frequent breaks due to cattle activity. Furthermore, in both sites there were Cerrado patches among pasture areas, riparian vegetation varied between 1 and 5 m in width, there were retention mechanisms in the water course, such as stones and trunks, stable banks with some cutting, riverbeds with silt, gravel, and sand, and in some sites, leaf detritus and woody material with sediments. The other seven streams were classified as preserved environments with HII varying from 0.81 to 0.96. At these points, the predominant characteristics were: more than 50 m in width of gallery forest, which was continuous with the adjacent forest, retention mechanisms strongly fixed, lack of banks, little or no accumulation of sediments in the riverbed, with stones grouped together, mosses and algae patches, and leaf detritus and woody material without sediment.

In general, the water streams of the Taquaruçu Grande watershed were well oxygenated, with low electrical conductivity, acid pH, and low concentration of Total Dissolved Solids (STD), except for streams Taq8 and Taq9 that showed high values of electrical conductivity and STD (Table 2).

A total of 615 individuals were collected that belong to nine orders and 30 families (Table 3). The order Trichoptera was present in high abundance with 187 individuals, followed by Coleptera with 145 individuals, and they occurred in all streams. Perlidae, the only taxa belonging to Plecoptera in the samples, was the most abundant family in the streams, and it was present in seven streams, but not in Taq9, with the lowest HII score. The family Elmidae from the order Coleoptera was the second most abundant in the streams, but it was absent from Taq9. Also, the family Hydropsychidae, an abundant trichopteran in the streams (62 individuals) presented a low abundance in Taq9, where only two individuals were captured. The order Lepidoptera was represented by one individual of the family Pyralidae, and it was the rarer group represented in the samplings and occurred only in Taq7 stream. Also rare, the order Megaloptera was represented only by 56 individuals of the family Corylidae, although it occurred in five among the nine sampled streams.

The Trichoptera occurred in all streams. The Ephemeroptera was absent from Taq5, but it occurred in all other streams. The Plecoptera occurred in all but two streams, Taq4 and Taq9. Also, Coleptera, Diptera, Lepidoptera, and Megaloptera were absent from Taq9. The anisopterans (Odonata) were present in Taq9 where they represented 29% of the Odonata or 2.76 of the abundance of macroinvertebrates in all samples.

Lepidoptera, Plecoptera, and Megaloptera were represented by one taxon each, whereas Coleoptera and Odonata were represented by seven and six taxa each.

The metrics based on macroinvertebrates fauna are presented in Table 4 and Table 5. According to the BMWP index from Monteiro et al. [21], only Taq7 and Taq8 streams had satisfactory environmental quality, and Taq3 was classified as having very poor quality. This index was strongly related to the abundance of macroinvertebrates, total richness, and richness of EPT (Table 6). Taq7 presented the greatest abundance and total richness of macroinvertebrates, which probably influenced its classification of being of “Good” environmental quality, whereas Taq3 presented the lowest abundance and EPT richness, which led to its “Very poor” classification (although it received a high HII score). Taq8 received a HII score equivalent to “Altered environment”, whereas it received a “Good quality” BMPW score. On the other hand, Taq1 presented a higher %EPT and EPT/total fauna ratio that was not correlated with the BMWP classification of “Poor quality” of this stream.

The metrics of the community (Shannon-Weiner index of diversity, Pielou index, and ratios of shredders and detritivores) did not correlate with the BMWP.

The BMWP index correlated positively to pH values, but not to other abiotic factors, including the Habitat Integrity Index (Table 7). The values of electrical conductivity and STD do not correlate with the BMWP index, since Taq8 showed “Good” environmental quality but presented altered values of those two measures. The other metrics based on macroinvertebrates fauna were not correlated with any physical, chemical, or structural characteristics of the environment.

## 4. Discussion

Impacts of anthropic intervention in watersheds in Cerrado are usually linked to urbanization and agriculture, and the studies on biological monitoring of those streams focus on the effects of organic and agricultural pollution on aquatic communities [11,32]. The present study examines a set of streams impacted by rural settlements and touristic activities. The main human interventions in the area are river deviation for landscape building and damming of rivers for recreational (bathing) purposes or water extraction for cattle or small-scale agriculture, together with annual fire management of Cerrado vegetation. Touristic activities are concentrated in the dry season and include habitat alteration due to excessive use, deterioration of water quality, and deforestation of spots in stream margins (bathing areas).

These activities produced low physical alterations in the streams courses, such as few exposed riverbanks, rare fragmentation and siltation points, and pasture surrounding the riparian vegetation, and so contributed to a high value for the physical Habitat Integrity Index. Although most of the sampled streams exhibit good physical, chemical, and structural (HII) quality, when considering the sensitivity of taxa expressed as BMWP scores, the sampled streams have generally poor quality. This is probably because long-term fluctuating disturbances of surrounding environments, such as fire disturbances, affect the insect fauna both as adults and larvae [33]. Nevertheless, fire management may produce long-term alteration in the diversity and abundance of macroinvertebrates that are probably reflected in the poor evaluation by BMWP. In a study of the responses of macroinvertebrate communities to fire via comparisons of streams in burned and unburned catchments in three fire-prone biomes that differ biogeographically and climatically (northwestern Mediterranean, southeastern Australia, and northwestern intermountain USA), Verkaik et al. [34] found that the responses of macroinvertebrate communities in streams in burned catchments were similar in all biogeographic regions and corresponded to reduced measures of taxonomic richness and increased abundance, especially of *r*-strategist taxa. Fire effects on streams generally result in long-term adverse changes in invertebrates leading to abundance and biomass decline and community composition shifts toward disturbance-adapted taxa [35,36]. It is possible that the vegetation in the Cerrado area studied recovers fast, but not the macroinvertebrate community, and the high HII scores do not account for the disturbance in the shifts of abundance and richness of sensitive families of macroinvertebrates as BMWP and EPT richness do.

All streams presented riparian forests; this vegetation directly influences the community structure of aquatic insects, mainly by the input of nutrients and allochthonous energy [37,38,39]. Sensitive taxa such as trichoperans and Odonata respond to variations in canopy cover [30]. Monteiro-Junior et al. [40] have shown that species richness of zygopterans is correlated positively with the integrity of riparian vegetation. In Taquaruçu Grande basin, zygopterans were collected in streams at Taq6 and Taq8, but not Taq9, showed the lowest HII score. On the other hand, anisopetrans were abundant and corresponded to 2.7% of the taxa present in Taq9. According to Monteiro-Junior et al. [40], a negative relationship is expected between environmental integrity and the richness of anisopetrans. We have shown that the abundance of this subgroup of Odonata rises in streams with low HII. The order Trichoptera was the most abundant, although the most represented family Leptoceridae was absent from Taq8 and Taq9, the streams with altered HII score and high STD measures. This indicates that trichopterans may respond to the physical disturbances measured by HII. Our results agree with the findings of Mazeika et al. [41] that EPT families are sensitive to stressors other than organic pollution, such as flow regimes and stream morphology. Also, Godoy et al. [42] showed that changes in the physical structure of smaller streams led to an increase in the unpredictability of community dynamics in smaller impacted streams of Cerrado.

The gravelly substrate that is abundant in the streams studied is an important source of resources to several macroinvertebrates, including those of the order Trichoptera [43,44,45], which may explain the higher abundance of this group in this basin.

Electrical conductivity and concentration of total dissolved solids, associated with anthropogenic impacts such as organic enrichment [46] and soil erosion [47], are two important variables in the structure of the aquatic community [46,48]. Measures of these two variables in two streams with high HII score (Taq8 and Taq9) were high, but there was no correlation with the biological metrics used. Taq8 was one of the two streams considered to have good environmental quality based on BMWP. These results could be a consequence of the predominant substrate in these streams, where the riverbed showed little accumulation of organic matter (considered a poor substrate), so that a small organic increment could contribute in favor of the fauna by acting as an intermediary disorder increasing taxa richness [49] and consequently the score BMWP in Taq8, but a more effective increment, similar to what had happened in Taq9 (based on the values of electrical conductivity and STD) could negatively influence the community.

Melo [50] showed that stream size and conductivity explained most of the variability in the macroinvertebrate community in a tropical stream. This is probably due to the fact that conductivity is as a general measure of disturbance, because it integrates the variables related to pollution, such as minerals and inorganic pollutants [51]. Although electrical conductivity was not correlated with BMWP index in Taquaruçu Grande streams, it is possible that a continuous monitoring may reveal the influence of conductivity in the macroinvertebrate fauna through temporal variation related to pulses of organic increments in the water of streams.

Only the pH of the Taquaruçu Grande streams was correlated to biotic metrics, and it was associated with increased BMWP. Thus, it is not possible to infer that environmental impacts of anthropization are influencing the community of aquatic macroinvertebrates by measuring solely abiotic parameters. The BMWP was correlated to three other metrics considered important for water monitoring, especially the richness of EPT [3] and richness of families [52]. In addition, the EPT fauna and the richness of families have been considered to be highly congruent with the community; in other words, they can be representative of the community diversity [32,53]. Thus, its information cannot be ignored despite the high abiotic quality of most of the streams, and it should be considered in the evaluation of environmental quality of aquatic ecosystems of low order in Northern Brazil’s Cerrado. The catchment area to which Taquaruçu Grande basin belongs is known as the medium Tocantins basin area with 46,307.31 ha, it has about one hundred streams of first order that correspond to the main tributaries, which demand fast and low cost but effective monitoring protocols. HII may be recommended as part of those protocols, since it partly reveals effects of disturbance to the aquatic communities. HII remains an inexpensive measure of riparian zone structural variables such as canopy openness, litter bank volume, number of retention devices, proportion of benthic substrate components, and water temperature [54], and should be considered for monitoring riparian deforestation in Northern Brazil’s Cerrado river ecosystems. The HII is directly related to the degree of environmental conservation and has been successfully used in other studies to evaluate the integrity in aquatic systems [55,56,57,58,59]. The HII, which is applied rapidly and easily, provides environmental managers with an objective measure of the degree of alteration of aquatic habitats [40].

Even though the Taquaruçu Grande is part of the Environmental Protection Area of Lajeado, it is suffering anthropic pressure by urbanization, agriculture, cattle farming, and tourism that negatively alter the integrity of the river ecosystems. Anthropic pressures bring significant loss of physical and chemical characteristics and therefore the reduction of aquatic biodiversity [5].

This study did not focus on the seasonal fluctuations of water flow in Cerrado streams. Northern Cerrado rivers are similar to Amazonia rivers in that they vary throughout the rainfall and dry period cycle [60]. Bleich et al. [54] found severe alterations in habitat structure (especially water temperature, oxygen, suspended materials, and nitrite concentrations) and availability of substrates (litter, trunks, and retention devices) in altered streams (measured by HII) in comparison to pristine ones. According to Pearson [61], current velocity is one of the main environmental correlates with species abundances and multivariate scores in tropical Australia. Faunal attributes cycled seasonally both in seasonal [61,62,63] and aseasonal [54,64] tropics.

Also, a single biotic index proved confusing in the case of the Taquaruçu Grande basin. BMWP is a biotic index focused on an organism’s tolerance to organic pollution [65,66], with the tolerance determined relative to levels based on dissolved oxygen [67,68,69]. However, Herman et al. [70] point out that this approach does not take into account the combined impacts of multiple stressors within streams or the complex nature of stream ecosystems. In addition, BMWP was created in Britain and adapted for other regions. In Brazil, Junqueira et al. [69] adapted BMWP for the Rio das Velhas basin, in the Southeast Brazil Cerrado area, and Monteiro et al. [21] for the Meia Ponte River, also in the Southeast hydrogeographic region of Paraná River in Brazil. Monteiro et al. [21] found different values for tolerance for the families: Elmidae, Hydrophilidae, Hydropsychidae, Leptoceridae, Odontoceridae, Leptophlebiidae, Baetidae, Perlidae, Gomphydae, Libellulidae, Calopterygidae, and Naucoridae, that are represented in the streams of Taquaruçu Grande. They may present a different value for tolerance in these streams when compared to the Meia Ponte basin streams, and further studies are needed to include local or regional values for tolerance in these families. It could also be recommended that other metrics should be included in a multi-metric index in order to better account for the complexity of stream ecosystems by way of a more comprehensive view of what is occurring within streams [71,72].

Also, according to Everaert et al. [73], the associations between macroinvertebrates and abiotic factors appear to be river-specific and therefore not automatically transferable between river basins in the tropics. Low BMWP values that do not correlate with the physical and chemical variables may indicate that communities of macroinvertebrates in the Taquaruçu Grande river basin do not show the same correlation with the abiotic parameters as those in the study by Monteiro et al. [21] in Meia Ponte River in the Cerrado of Goiás State, Brazil. This highlights the importance of establishing knowledge on the macroinvertebrate responses to alterations in different stream ecosystems in Cerrado. The Taquaruçu Grande basin is exemplary of the Northern Cerrado transition ecosystems that differ from the Cerrado of Goiás, which are more similar to the core of Southeast Brazil’s Cerrado landscapes. Although there is no detailed geographic classification of Brazilian ecoregions [74], it is widely known that the Cerrado biome is a mosaic of different vegetation ecosystems and the Taquaruçu Grande basin is in the core of a vast transition zone of Cerrado with the biomes Caatinga and Amazonia. Ligeiro et al. [74] point out that reference sites should be specific for a particular typology (e.g., altitude, stream size, and predominant substrate) and geographic domain (biome and ecoregion). As human modifications are widespread and occur across most landscapes on a worldwide basis, sites in least-disturbed condition may be used to represent reference conditions [75,76,77]. This is the first study on the macroinvertebrate fauna in these ecosystems, and it raises the question as to whether studies in Cerrado ecosystems in the Southeast and West Brazil can model the parameters for biomonitoring of river ecosystems in the Northern part of this threatened biome.

## 5. Conclusions

The macroinvertebrates in the Northern Cerrado streams from Taquaruçu Grande Basin present different responses to environmental variables. Trichopterans and Odonata families show a positive response to physical aspects of the riparian zone, as measured by HII. On the other hand, BMWP scores were strongly correlated with other biotic metrics, such as richness of EPT and richness of families, but neither to HII nor to chemical parameters of the water. This seems to indicate that persistent disturbance in the streams may be due to annual fire management and ecotouristic activities in the dry season. Single parameters, especially conductivity and dissolved solids, seem to reflect well the physical degradation of riparian zones as expressed by low HII scores for two streams, but did not correlate with biotic metrics. This may be partially derived from a single collection effort that did not consider the seasonality of the flow regime in the region. Through the use of biotic metrics based on the macroinvertebrate fauna, this study revealed that the streams are suffering from environmental impacts. These impacts are believed to be linked to annual events of disturbance that cause the reduction and modification of sensitive taxa, although the study demonstrated that the alterations to the local macroinvertebrate communities were not immediately detectable by measures of abiotic variables. A sole index could not completely characterize the degree of pristinity or disturbance to the stream ecosystems, and, as such, longer monitoring and new metrics must be tested for the Northern Cerrado river systems.

## Figures and Tables

**Table 1:** Physical and chemical characterization of nine streams in the Taquaruçu Grande river basin, State of Tocantins, Brazil.

Stream	Order	Channel Extension (m)	Width (cm)	Depth (cm)	Riparian Cover		
					Width	Type	Surrounding Cover Matrix
Taq1	1	5872.78	37	10	>100 m	Continuous forest	Dense Cerrado vegetation
Taq2	1	311.28	27	10	30–100 m	Continuous forest	Dense Cerrado vegetation
Taq3	1	1155.64	40	6	>100 m	Continuous forest	Dense Cerrado vegetation
Taq4	1	630.63	54	5	5–30 m	Continuous forest	Cerrado savanna (herbaceous)
Taq5	2	1674.35	90	28	1–5 m	Open Cerrado forest	Cerrado savanna (herbaceous)
Taq6	1	648.22	44	8	>100 m	Continuous forest	Dense Cerrado vegetation
Taq7	2	6304.74	170	22	5–30 m	Continuous forest	Pasture
Taq8	2	2942.65	100	12	1–5 m	Open savanna (shrubs)	Herbaceous vegetation with sparse shrubs
Taq9	2	6455.55	263	44	1–5 m	Open savanna (shrubs)	Herbaceous vegetation with sparse shrubs

**Table 2:** Physical and chemical characterization of nine streams in the Taquaruçu Grande river basin, State of Tocantins, Brazil. HII, Index of Habitat Integrity. OD = Dissolved Oxygen. STD = Total Dissolved Solids.

Stream	Discharge (m^3^/s)	Temp. (°C)	OD (mg/L)	pH	C (µS/cm)	STD (ppm)	HII
Taq1	0.0028	24.7	8.48	5.28	20.0	10.0	0.92
Taq2	0.0014	25.6	7.29	5.49	20.0	10.0	0.93
Taq3	0.0012	23.1	8.06	4.90	20.0	10.0	0.92
Taq4	0.0007	22.1	8.04	5.51	20.0	18.0	0.89
Taq5	0.0188	23.2	8.25	5.92	10.0	10.0	0.88
Taq6	0.0012	22.6	8.25	5.72	20.0	12.0	0.96
Taq7	0.1271	22.8	8.22	5.89	20.0	10.0	0.81
Taq8	0.0150	30.7	6.60	6.29	62.0	30.4	0.77
Taq9	0.2038	24.9	8.54	5.69	90.0	60.0	0.56
Standard deviation	0.401	26.476	16.829	0.073	78.337	12.92	*

* Not calculated because this is a composite index. Note: Data is the mean of 10 measures in each stream.

**Table 3:** Families of aquatic macroinvertebrates collected in nine streams at Taquaruçu Grande river basin, State of Tocantins, Brazil.

Taxa	Frequency of Occurrence (%)		Relative Abundance (%)
Coleoptera	Elmidae	67	13.01
	Dryopidae	44	7.64
	Noteridae	33	1.30
	Ptylodactylidae	22	0.49
	Dytiscidae	11	0.81
	Hydrophilidae	11	0.16
	Gyrinidae	11	0.16
Diptera	Simuliidae	44	4.07
	Chironomidae	44	1.95
Lepidoptera	Pyralidae	11	0.16
Trichoptera	Hydropsychidae	67	10.08
	Leptoceridae	56	15.93
	Odontoceridae	44	1.63
	Calamoceratidae	33	2.76
Ephemeroptera	Leptophlebiidae	56	1.95
	Baetidae	44	7.84
	Euthyplociidae	11	0.49
Plecoptera	Perlidae	78	10.40
Odonata	Gomphidae	56	5.37
	Libellulidae	44	1.79
	Perilestidae	22	0.81
	Polythoridae	22	0.49
	Calopterygidae	11	0.81
	Coenagrionidae	11	0.33
Hemiptera	Naucoridae	11	0.81
	Veliidae	44	0.81
	Belostomatidae	33	0.81
	Notonectidae	33	0.65
	Gerridae	11	0.49
Megaloptera	Corydalidae	56	1.63

**Table 4:** Results of Biological Monitoring Working Party (BMWP) [21] applied to nine streams of the Taquaruçu Grande river basin, State of Tocantins, Brazil.

Stream	BMWP	BMWP Classification
Taq1	43	Poor
Taq2	46	Poor
Taq3	19	Very poor
Taq4	36	Poor
Taq5	37	Poor
Taq6	44	Poor
Taq7	97	Good
Taq8	77	Good
Taq9	31	Poor

**Table 5:** Biological metrics of the macroinvertebrate fauna for the nine streams of Taquaruçu Grande river basin, State of Tocantins, Brazil. H: Shannon-Weiner index of diversity. J: Pielou index. EPT: Ephemeroptera, Plecoptera, and Trichoptera.

Stream	Abundance	H	J	Total Richness	EPT Richness	EPT (%)	Ratio of EPT/Total	Ratio of Shredders	Ratio of Detritivores
Taq1	77	1.5	0.66	9	3	80.52	80.52	0.00	1.30
Taq2	115	1.71	0.69	12	6	37.39	37.39	39.13	0.87
Taq3	19	1.57	0.81	7	2	47.37	47.37	36.84	0.00
Taq4	30	2.37	0.90	14	4	43.33	43.33	23.33	16.67
Taq5	65	1.62	0.83	7	3	52.31	52.31	1.54	0.00
Taq6	43	2.01	0.81	12	3	53.49	53.49	4.65	6.98
Taq7	169	2.10	0.74	17	6	55.03	55.03	0.59	2.37
Taq8	69	2.34	0.84	16	5	23.19	23.19	7.25	10.14
Taq9	28	1.53	0.79	7	3	32.14	32.14	0.00	10.71

**Table 6:** Correlations of BMWP with other studied biotic metrics based on macroinvertebrate fauna of nine streams of Taquaruçu Grande river basin, State of Tocantins, Brazil.

Metrics		Abundance	H	J	Total Richness	EPT Richness	EPT (%)	Ratio of EPT/Total	Ratio of Shredders	Ratio of Detritivores
**BMWP-Monteiro et al. (2008) [21] **	r	0.808	0.550	−0.160	0.836	0.775	−0.077	−0.077	−0.384	0.002
	*p*	0.008	0.125	0.680	0.005	0.014	0.844	0.844	0.308	0.995

Note: Significant values in bold at 0.05. r = Pearson correlation, *p* = test of significance.

**Table 7:** Correlations between metrics based on macroinvertebrate fauna and abiotic data, including the Habitat Integrity Index for nine streams of Taquaruçu Grande river basin, State of Tocantins, Brazil.

Metrics		BMWP-Monteiro et al. (2008) [21]	Ratio of Shredders	Ratio of Detritivores	Ratio of EPT/Total	Abundance	EPT (%)	H	J	Total Richness	EPT Richness
**Temperature**	r	0.348	−0.063	0.123	−0.525	0.082	−0.525	0.182	−0.070	0.263	0.336
	p	0.359	0.873	0.753	0.146	0.834	0.146	0.639	0.858	0.495	0.376
**Dissolved Oxygen**	r	−0.379	−0.366	−0.134	0.642	−0.170	0.642	−0.505	−0.143	−0.523	−0.555
	p	0.315	0.332	0.732	0.063	0.662	0.063	0.166	0.713	0.149	0.121
**pH**	r	0.669	−0.572	0.308	−0.430	0.336	−0.430	0.541	0.285	0.528	0.487
	p	0.049	0.108	0.421	0.249	0.376	0.249	0.133	0.457	0.144	0.184
**Total dissolved solids**	r	−0.106	−0.308	0.562	−0.571	−0.364	−0.571	−0.042	0.213	−0.170	−0.116
	p	0.787	0.420	0.115	0.108	0.336	0.108	0.914	0.583	0.662	0.767
**Electrical conductivity**	r	0.031	−0.291	0.491	−0.608	−0.271	−0.608	−0.003	0.125	−0.063	−0.022
	p	0.936	0.447	0.180	0.082	0.481	0.082	0.994	0.749	0.872	0.956
**Discharge**	r	0.212	−0.460	0.182	−0.234	0.159	−0.234	−0.162	−0.088	−0.061	0.099
	p	0.583	0.213	0.640	0.544	0.682	0.544	0.678	0.821	0.875	0.800
**HII**	r	−0.128	0.412	−0.398	0.512	0.088	0.512	0.051	−0.120	0.077	−0.045
	p	0.743	0.271	0.289	0.158	0.823	0.158	0.895	0.759	0.844	0.908

## References

[1] Vorosmarty C.J., McIntyre P.B., Gessner M.O., Dudgeon D., Prusevich A., Green P., Glidden S., Bunn S.E., Sullivan C.A., Liermann C.R. (2010). Rivers in Crisis: Global Water Insecurity for Humans and Biodiversity. Nature.

[2] Allan J.D. (2004). Landscapes and rivers capes, the influence of land use on stream ecosystems. Annu. Rev. Ecol. Evol. Syst..

[3] Laurance W.F., Sayer J., Cassman K.G. (2014). Agricultural expansion and its impacts in tropical nature. Trends Ecol. Evol..

[4] Casatti L., Langeani F., Silva A.M., Castro R.M.C. (2006). Stream fish, water and habitat quality in a pasture dominated basin, southeastern Brazil. Braz. J. Biol..

[5] Allan J.D., Flecker A.S. (1993). Biodiversity conservation in running waters. BioScience.

[6] Myers N., Mittermeier R.A., Mittermeier C.G., Fonseca G.A.B., Kent J. (2000). Biodiversity hotspots for conservation priorities. Nature.

[7] Clement C.R., Higuchi N. (2006). A floresta amazônica e o futuro do Brasil. Cienc. Cult..

[8] Trancoso R., Carneiro Filho A., Tomasella J., Schietti J., Forsberg B.R., Miller R.P. (2009). Deforestation and conservation in major watersheds of the Brazilian Amazon. Environ. Conserv..

[9] Hayhoe S.J., Neill C., Porder S., Mchorney R., Lefebvre P., Coe M.T., Elsenbeer H., Krusche A.V. (2011). Conversion to soy on the Amazonian agricultural frontier increases streamflow without affecting stormflow dynamics. Glob. Chang. Biol..

[10] Meyer J.L., Strayer D.L., Wallace J.B., Eggert S.L., Helfman G.S., Leonard N.E. (2007). The contribution of headwater streams to biodiversity in river networks. J. Am. Water Resour. Assoc..

[11] Coe M.T., Costa M.H., Soares-Filho B.S. (2009). The influence of historical and potential future deforestation on the stream flow of the Amazon River—Land surface processes and atmospheric feedbacks. J. Hydrol..

[12] Chaves J., Neill C., Germer S., Gouveia Neto S., Krusche A., Elsenbeer H. (2008). Land management impacts on runoff sources in small Amazon watersheds. Hydrol. Process..

[13] Germer S., Neill C., Krusche A.V., Elsenbeer H. (2010). Influence of land-use change on near-surface hydrological processes: Undisturbed forest to pasture. J. Hydrol..

[14] Deegan L.A., Neill C., Haupert C.L., Ballester M.V.R., Krusche A.V., Victoria R.L., Thomas S.M., Moor E. (2011). Amazon deforestation alters small stream structure, nitrogen biogeochemistry and connectivity to larger rivers. Biogeochemistry.

[15] Rosenberg D.M., Resh V.H. (1993). Introduction to freshwater biomonitoring and benthic macroinvertebrates. Freshwater Biomonitoring and Benthic Macroinvertebrates.

[16] Bonada N., Prat N., Resh V.H., Statzner B. (2006). Developments in aquatic insect biomonitoring: A comparative analysis of recent approaches. Annu. Rev. Entomol..

[17] Callisto M., Gonçalves J.F., Moreno P. (2004). Invertebrados aquáticos como bioindicadores. Navegando o Rio das Velhas das Minas aos Gerais.

[18] Moreno P., Callisto M., Silveira M. (2006). Bioindicadores de qualidade de água ao longo da bacia do Rio das Velhas (MG). Bioindicadores de Qualidade de Água, Capítulo 5.

[19] Andrade H.T.A., Santiago A.S., Medeiros J.F. (2008). Estrutura da Comunidade de Invertebrados Bentônicos com Enfoque nos Insetos Aquáticos do Rio Piranhas-Assu, Rio Grande do Norte, Nordeste do Brasil. Entomo Bras..

[20] Carrera C., Fierro K. (2001). Manual de Monitoreo: Los Macroinveretebrados Acuáticos Como Indicadores de la Calidad del Agua.

[21] Monteiro T.R., Oliveira L.G., Godoy B.S. (2008). Biomonitoramento da qualidade da água utilizando macroinvertebrados bentônicos: Adaptação do Índice Biótico BMWP à Bacia do Rio Meia Ponte-GO. Oecol. Bras..

[22] Martins J.C., Costa J.C. (2009). Os Macroinvertebrados no Ensino da Biologia.

[23] Ranzani G. (2002). Solos e Aptidão Agrícola das Terras do Município de Palmas—Tocantins.

[24] Embrapa (2000). Levantamento Semidetalhado dos Solos na Bacia do Olaria-DF.

[25] Tocantins (Estado) (2012). Secretaria de Planejamento e Meio Ambiente. Diretoria de Zoneamento Ecológico-Econômico. Atlas do Tocantins: Subsídios ao Planejamento da Gestão Territorial.

[26] Foundation University of Tocantins (1999). Plano de Manejo da Sub-Bacia do Ribeirão Taquaruçu Grande—(SOS Taquaruçu).

[27] Pes A.M.O., Hamada N., Nessimian J.L. (2005). Chaves de identificação de larvas para famílias e gêneros de Trichoptera (Insecta) da Amazônia Central, Brasil. Rev. Bras. Entomol..

[28] Hamada N., Ferreira-Keppler R.L. (2012). Guia Ilustrado de Insetos Aquáticos e Semiaquáticos da Reserva Florestal Ducke.

[29] Nessimian J.L., Venticinque E.M., Zuanon J., de Marco P., Gordo M., Fidelis L., Batista J.D., Juen L. (2008). Land use, habitat integrity and aquatic insect assemblages in Central Amazonian streams. Hydrobiologia.

[30] Pereira L.R., Cabette H.S.R., Juen L. (2012). Trichoptera as bioindicators of habitat integrity in the Pindaíba River basin, Mato Grosso (Central Brazil). Ann. Limnol. Int. J. Limnol..

[31] Cummins K.W., Merritt R., Andrade P. (2015). The use of invertebrate functional groups to characterize ecosystem attributes in selected streams and rivers in south Brazil. Stud. Neotrop. Fauna Environ..

[32] Ferreira W.R., Paiva L.T., Callisto M. (2011). Development of a benthic multimetric index for biomonitoring of a neotropical watershed. Braz. J. Biol..

[33] Malison R.L., Baxter C.V. (2010). Streams affected by wildfire of varying severity differ in aquatic insect assemblage structure and emergence. J. N. Am. Benthol. Soc..

[34] Verkaik I., Vila-Escalé M., Rieradevall M., Baxter C.V., Lake P.S., Minshall G.W., Reich P., Prat N. (2015). Stream macroinvertebrate community responses to fire: Are they the same in different fire-prone biogeographic regions?. Freshw. Sci..

[35] Rugenski A.T., Minshall G.W. (2014). Climate-moderated responses to wildfire by macroinvertebrates and basal food resources in montane wilderness streams. Ecosphere.

[36] Verkaik I., Rieradevall M., Cooper S.D., Melack J.M., Dudley T.L., Prat N. (2013). Fire as a disturbance in Mediterranean climate streams. Hydrobiologia.

[37] Gonçalves-Junior J.F., França J.S., Medeiros A.O., Rosa C.A., Callisto M. (2006). Leaf breakdown in a tropical stream. Int. Rev. Hydrobiol..

[38] Gonçalves-Junior J.F., Graça M.A.S., Callisto M. (2006). Leaf-litter breakdown in 3 streams in temperate, Mediterranean, and tropical Cerrado climates. J. N. Am. Benthol. Soc..

[39] Jacobsen D., Cressa C., Mathooko J.M., Dudgeon D., Dudgeon D. (2008). Macroinvertebrates: Composition, life histories and production. Tropical Stream Ecology.

[40] Monteiro-Junior C.S., Juen L., Hamada N. (2014). Effects of urbanization on stream habitats and associated adult dragonfly and damnselfly communities in central Brazilian Amazonia. Landsc. Urban Plan..

[41] Sullivan S.M., Watzin M.C., Hession W.C. (2004). Understanding stream geomorphic state in relation to integrity: Evidence using habitat assessment and macroinvertebrates. Environ. Manag..

[42] Godoy B.S., Simião-Ferreira S., Lodi S., Oliveira L.G. (2016). Functional processing zones characterizing aquatic insetc communities in streams of Brazilian Cerrado. Noetrop. Entomol..

[43] Duan X., Wang Z., Tian S. (2008). Effect of streambed substrate on macroinvertebrate biodiversity. Front. Environ. Sci. Eng. China.

[44] Flecker A.S., Allan J.D. (1984). The importance of predation, substrate and spatial refugia in determining lotic insect distributions. Oecologia.

[45] Kikuchi R.M., Uieda V.S., Nessimian J.L., Carvalho A.L. (1998). Composição da comunidade de invertebrados de um ambiente lótico tropical e sua variação espacial e temporal. Ecologia de Insetos Aquáticos.

[46] Couceiro S.R.M., Hamada N., Luz S.L.B., Forsberg B.R., Pimentel T.P. (2007). Deforestation and sewage effects on aquatic macroinvertebrates in urban streams in Manaus, Amazonas, Brazil. Hydrobiologia.

[47] Nascimento B.L.M., de Souza Gomes D.R.C., Costa G.P., Araújo S.S., dos Santos L.C.A., de Oliveira J.D. (2015). Comportamento e avaliação de metais potencialmente tóxicos (Cu(II), Cr(III), Pb(II) e Fe(III)) em águas superficiais dos Riachos Capivara e Bacuri Imperatriz-MA, Brasil. Eng. Sanit. Ambient..

[48] Couceiro S.R.M., Hamada N., Forsberg B.R., Padovesi-Fonseca C. (2010). Effects of anthropogenic silt on aquatic macroinvertebrates and abiotic variables in streams in the *Brazilian Amazon*. J. Soils Sediments.

[49] Connel J.H. (1978). Diversity in tropical rain forest and coral reefs. Science.

[50] Melo A.S. (2009). Explaining dissimilarities in macroinvertebrate assemblages among stream sites using environmental variables. Zoologia.

[51] D’heygere T., Goethals P.L.M., de Pauw N. (2003). Use of genetic algorithms to select input variables in decision tree models for the prediction of benthic macroinvertebrates. Ecol. Model..

[52] Couceiro S.R.M., Hamada N., Forsberg B.R., Pimentel T.P., Luz S.L.B. (2012). A macroinvertebrate multimetric index to evaluate the biological condition of streams in the Central Amazon region of Brazil. Ecol. Indic..

[53] Heino J., Mykrä H., Kotanen J., Muotka T. (2007). Ecological filters and variability in stream macroinvertebrate communities: Do taxonomic and functional structure follow the same path?. Ecography.

[54] Bleich M.E., Mortati A.F., André T., Piedade M.T.F. (2014). Riparian deforestationaffects the structural dynamics of headwater streams in Southern Brazilian Amazonia. Trop. Conserv. Sci..

[55] Dias-Silva K., Cabette H.S.R., Juen L., Marco Junior P. (2010). The influence of habitat integrity and physical-chemical water variables on the structure of aquatic and semi-aquatic Heteroptera. Zoologia.

[56] Juen L., Marco Junior P. (2011). Odonate biodiversity interra-firme forest streamlets in Central Amazonia: On the relative effects of neutral and niche drivers at small geographical extents. Insect Conserv. Divers..

[57] Nogueira E.M., Fearnside P.M., Nelson B.W., França M.B. (2007). Wood density in forests of Brazil’s ‘arc of deforestation’: Implications for biomass and flux of carbon from land-use change in Amazonia. For. Ecol. Manag..

[58] Shimano Y., Cabette H.S.R., Salles F.F., Juen L. (2010). Composição e distribuição da fauna de Ephemeroptera (Insecta) em área de transição Cerrado-Amazônia, Brasil. Iheringia Sér. Zool..

[59] Souza H.M.L., Cabette H.S.R., Juen L. (2011). Baetidae (Insecta, Ephemeroptera) em córregos do cerrado matogrossense sob diferentes níveis de preservação ambiental. Iheringia Sér. Zool..

[60] Junk W.J., Bayley P.B., Sparks R.E. (1989). The flood pulse concept in river-floodplain-systems. Can. J. Fish. Aquat. Sci..

[61] Pearson R.G. (2014). Dynamics of invertebrate diversity in a tropical system. Diversity.

[62] Leung A.S.L., Dudgeon D. (2011). Scales of spatiotemporal variability in macroinvertebrate abundance and diversity in monsoonal streams: Detecting environmental change. Freshw. Biol..

[63] Leung A.S.L., Li A.O.Y., Dudgeon D. (2012). Scales of spatiotemporal variation in macroinvertebrate assemblage structure in monsoonal streams: The importance of season. Freshw. Biol..

[64] Rawi C.S.M., Al-Shami S.A., Madrus M.R., Ahmad A.H. (2014). Biological and ecological diversity of aquatic macroinvertebrates in response to hydrological and physicochemical parameters in tropical forest streams of Gunung Tebu, Malaysia: Implications for ecohydrological assessment. Ecohydrology.

[65] Hilsenhoff W.L. (1987). An improved biotic index of organic stream pollution. Great Lakes Entomol..

[66] Ollis D.J., Dallas H.F., Esler K.J., Boucher C. (2006). Bioassessment of the ecological integrity of river ecosystems using aquatic macroinvertebrates: An overview with a focus on South Africa. Afr. J. Aquat. Sci..

[67] Chesters K.R. (1980). Biological Monitoring Working Party: The 1978 National Testing Exercise.

[68] Hawkes H.A. (1998). Origin and development of the biological monitoring working party score system. Water Res..

[69] Junqueira V.M., Campos S.C.M. (1998). Adaptation of the BMWP method for water quality evaluation to Rio das Velhas watershed (Minas Gerais, Brazil). Acta Limnol. Bras..

[70] Herman M.R., Nejadahshemi A.P. (2015). A review of macroinvertebrate- and fish-based stream health indices. Ecohidrol. Hydrobiol..

[71] Thorne R., Williams P. (1997). The response of benthic macroinvertebrates to pollution in developing countries: A multimetric system of bioassessment. Freshw. Biol..

[72] Rakocinski C.F. (2012). Evaluating macrobenthic process indicators in relation to organic enrichment and hypoxia. Ecol. Indic..

[73] Everaert G., de Neve J., Boets P., Dominguez-Granda L., Mereta S.T., Ambelu A., Hoang T.H., Goethals P.L.M., Thas O. (2014). Comparison of the Abiotic Preferences of Macroinvertebrates in Tropical River Basins. PLoS ONE.

[74] Ligeiro R., Highes R.M., Kaufmman P.R., Macedo D.R., Firmiciano K.R., Ferreira W.R., Oliveira D., Melo A.S., Callisto M. (2013). Defining quantitative stream disturbance gradients and the additive role of habitat variation to explain macroinvertebrate taxa richness. Ecol. Indic..

[75] Reynoldson T.B., Norris R.H., Resh V.H., Day K.E., Rosenberg D.M. (1997). The reference condition: A comparison of multimetric and multivariate approaches to assess water quality impairment using benthic macroinvertebrates. J. N. Am. Benthol. Soc..

[76] Stoddard J., Larsen D.P., Hawkins C.P., Johnson R.K., Norris R.H. (2006). Setting expectations for the ecological condition of streams: The concept of reference condition. Ecol. Appl..

[77] Yates A.G., Bailey R.C. (2010). Selecting objectively defined reference sites for stream bioassessment programs. Environ. Monit. Assess..

